# Complete Genome Sequence of *Streptomyces* sp. Sge12, Which Produces Antibacterial and Fungicidal Activities

**DOI:** 10.1128/genomeA.00415-17

**Published:** 2017-05-25

**Authors:** Jianguo Xu, Min Xu, Kai Liu, Qinyin Peng, Meifeng Tao

**Affiliations:** State Key Laboratory of Microbial Metabolism, School of Life Sciences and Biotechnology, Shanghai Jiao Tong University, Shanghai, China

## Abstract

*Streptomyces* sp. Sge12 was isolated from forest soil and exhibited remarkable antimicrobial activities against selected fungi and Gram-positive bacteria. Here, we report the complete genome sequence of this strain, which contains 37 putative secondary metabolite gene clusters.

## GENOME ANNOUNCEMENT

*Streptomyces* is the largest genus of *Actinobacteria*, and its members are the major targets of genome mining for the discovery of bioactive secondary metabolites (1). *Streptomyces* sp. strain Sge12 was isolated from the forest soil of Shengnongjia, Northwest Hubei Province, China, and was preserved at the China Center for Type Culture Collection (CCTCC AA92011). *Streptomyces* sp. Sge12 cultured on YBP agar (2) exhibited strong growth-inhibitory activities against selected Gram-positive bacteria, including *Staphylococcus aureus*, *Mycobacterium smegmatis* mc^2^155, and *Bacillus mycoides*, and fungi such as *Gibberella zeae* and *Thanatephorus cucumeris*. Here, we report the sequenced genome of *Streptomyces* sp. Sge12 in order to analyze its potential for the mining of novel bioactive secondary metabolites.

The whole genome of *Streptomyces* sp. Sge12 was sequenced by using a combined strategy of paired-end Illumina HiSeq 4000 (688.0-Mb sequences, 84.8-fold coverage) and single-molecule real-time PacBio RSII (1,127.1-Mb sequences, 139.0-fold coverage) sequencing. The reads from the Illumina HiSeq 4000 and PacBio RSII sequencing were assembled using Meraculous version 2.0 software (3) and RS_HGAP Assembly version 3.0 software (4), respectively. Subsequently, the final assembly of the whole genome was finished by using Illumina/PacBio hybrid assembly approaches (5). The total size of the genome is 8,110,698 bp, with a G+C content of 72.17%, and contains a linear chromosome (7,983,613 bp) and a circular plasmid (pSGE, 127,085 bp). Next, open reading frames of the genome were predicted by Glimmer version 3.02, and the predicted genes were annotated using the NR, COG, GO, Swiss-Prot, and KEGG databases. In general, the whole genome encompasses 7,491 protein-coding genes (with 126 of them located on pSGE), 71 tRNA operons, and 21 rRNA operons.

The genome sequence of *Streptomyces* sp. Sge12 was examined using antiSMASH version 3.0 (6), leading to the identification of 37 putative biosynthetic gene clusters (BGCs) for various types of secondary metabolites, including 6 terpenes, 5 nonribosomal peptides, 5 polyketides, 3 nonribosomal peptide-polyketide hybrids, and 3 ribosomally synthesized and posttranslationally modified peptides (RiPPs), which may be involved in the observed antimicrobial activities. Only four exhibited 100% similarity with known gene clusters, which were responsible for the biosynthesis of the odorous metabolite 2-methylisoborneol (7), the siderophore desferrioxamine B (8), the morphogen SapB (9), and the compatible solute ectoine (10). An additional four gene clusters showed >50% similarity with the BGCs of hopene (11, 12), gray spore pigment (13), lactazole A (14), and alkylresorcinol (15). None of the above eight compounds has been reported to exhibit strong antimicrobial activity. Further experimental studies of the *Streptomyces* sp. Sge12 BGCs may lead to the discovery of antimicrobial metabolites and provide insights into their production.

### Accession number(s).

The genome sequence of *Streptomyces* sp. Sge12 has been deposited in the DDBJ/ENA/GenBank database under the GenBank accession numbers CP020555 for the linear chromosome and CP020556 for the circular plasmid pSGE (two entries).
